# Crossbreeding and consanguinity management in pig farms in the departments of Ouémé and Plateau in Benin

**DOI:** 10.14202/vetworld.2019.1816-1825

**Published:** 2019-11-21

**Authors:** Ignace Ogoudanan Dotché, Simon Idohou, Mahamadou Dahouda, Pascal Kiki, Benoit Govoeyi, Nicolas Antoine-Moussiaux, Jean-Paul Dehoux, Guy Apollinaire Mensah, Souaïbou Farougou, Pierre Thilmant, Issaka Youssao Abdou Karim, Benoît Koutinhouin

**Affiliations:** 1 Laboratory of Animal Biotechnology and Meat Technology, Department of Animal Production and Health, Polytechnic School of Abomey-Calavi, University of Abomey-Calavi, 01 BP 2009, Cotonou, Benin; 2 Department of Animal Production, Faculty of Agronomic Science, University of Abomey-Calavi, 01 BP 526, Cotonou, Benin; 3 Fundamental and Applied Research for Animals and Health, Faculty of Veterinary Medicine, University of Liège, Vallée 2, Avenue de Cureghem, B-4000 Liège, Belgium; 4 Laboratory of Experimental Surgery, Universite Catholique de Louvain, 55/70, Avenue Hippocrate, 1200, Brussels, Belgium; 5 Agricultural Research Center of Agonkamey, National Institute of Agricultural Research of Benin, 01 BP 884, Cotonou, Benin; 6 Department of Animal Production and Health, Polytechnic School of Abomey-Calavi, University of Abomey-Calavi, 01 BP 2009, Cotonou, Benin; 7 Provincial Center of Productions Animales, Liège (CPL Animal), Rue de Saint Remy, 5 B4601 Argenteau, Belgium

**Keywords:** consanguinity, crossbreeding, pig, zootechnical performances

## Abstract

**Background:**

The improvement in pig zootechnical performances is a common practice in Benin. This improvement of the performances is made by the choice of the best reproducers in farms and the crossbreeding between the different breeds.

**Aim:**

This study aims to characterize practices related to consanguinity management in pigs reared in Ouémé and Plateau.

**Materials and Methods:**

Crossbreeding and consanguinity data were collected from 60 farms in these two departments. Frequencies and averages were calculated and compared between departments, genetic types, and origin of progenitors.

**Results:**

The majority of the investigated pig farmers in both departments were married men of primary or secondary education level. Most of them cross animals without a specific crossbreeding scheme. These crossings were performed to a greater extent (p<0.05) in Ouémé (94.28%) than in Plateau (52%). In general, farmers cross improved animals of high breeding values with the crossbred ones. These crossings were mainly performed to improve zootechnical performances. Renewing animals were commonly chosen from the farm or were provided from nearby farms. The majority of pig breeders in Ouémé (100%) and Plateau (86.67%) obtained reproductive animals from nearby farms. Males and females were sometimes bought from the same farm or from farms that pig breeders have sold reproductive animals in the previous years. In the case of selection within their own farm, male and female progenitors are separated at puberty by the majority of the breeders of Plateau (42.11%) and Ouéme (50%). Inbred mating was reported by breeders. More than half of breeders mate animals having a parental link in both departments. The mating was performed between animals of the same mother in 37.93% of farms in Ouémé and in 45.46% in Plateau. The main consanguinity consequences mentioned by the breeders were the high mortality at birth and weaning, piglets’ weakness at the birth, the slow growth, and the decrease in litter size. Sows with at least one parent from external farm had a litter size at birth and weaning and a live-born piglets’ number significantly higher than sows with both parents from the same farm.

**Conclusion:**

Rigorous monitoring of crossing and the filial links are necessary for pig farms for ensuring the improvement of zootechnical performances.

## Introduction

The performance improvement is the common goal in farms in general and in pig farms in particular. This improvement is achieved through the crossing and selection of best progenitors. A well-monitored pig breeding improvement in Benin has already been carried out at a research station and is focused on the increase of local pigs’ zootechnical performances by crossing with imported high producing breeds [1]. These crossings have resulted in F1 hybrid animals whose reproduction and production performances were superior to those of local pigs [1]. After high-producing pigs (Large White, Landrace, and Meishan) introduction in Benin in 2004, by the Livestock Development Project [2], breeders crossed local breeds with these breeds. However, the lack of monitoring during these crossings by livestock services and the poor recording of zootechnical data have led to a lack of useful information for the evaluation of the different crossing patterns. These different crossings have led to a diversity of porcine genetic resources in farms grouped into three genetic types: Improved, local, and crossbred [3].

Despite these improvement efforts, reproduction and production performances of reared pigs are low [4] and do not allow domestic pork production to meet the population needs. To improve animals’ productivity, reproducers selection criteria have been assessed to allow breeders selecting animals according to their farm typology and production objectives and achieving significant genetic progress [5]. Due to the lack of alternatives, progenitors are often selected from the same farm and this practice results in increased rates of consanguinity since some breeders do not pay attention to the parental relationship when choosing a male for reproduction [5]. The consanguinity risk is high because farm size is too small [6] and the progenitors are often kept for long time in farms due to their high performance. Some farmers borrow boars from neighboring farms for their sows’ fertilization [7]. The way boars are used coupled with the random crossings between breeds pose risks of local breed extinction, open doors to consanguinity, and threatens genetic diversity [8]. Efficient management of improvement practices would allow breeders to benefit from the heterosis effect resulting from crossings between breeds instead of the depressive effect on the reproduction and production performances caused by the consanguinity [9].

This study aimed to improve the productivity of pigs reared in Benin by describing data related to the management of crossbreeding and consanguinity. Specifically, it aims to: (a) Characterize the modes of crossbreeding performed in pigs’ farms and (b) evaluate the effects of the consanguinity on the zootechnical performances of pigs on a farm level.

## Materials and Methods

### Ethical approval

The manuscript does not contain clinical studies or patient data, Ethical Committee approval was not required.

### Study area

**Figure-1:**
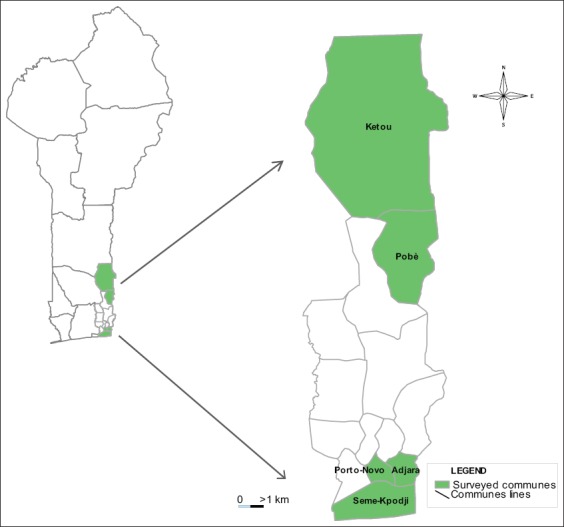
Study area on the crossbreeding and consanguinity management in pig farms in the departments of Ouémé and Plateau. Source: Manifold software and the base maps of DIVA-GIS (Available on: https://www.diva-gis.org/datadown).

Data were collected in the department of Ouémé and Plateau from May 2017 to September 2017. The department of Ouémé is located between 6° 40 ‘0 “Latitude North and 2° 30’ 0” East Longitude and covers an area of 1281 km² (1.12% of the national territory) with a population of 1,100,404 inhabitants [10]. Data were collected in the townships of: Adjarra, Porto-Novo, and Sèmè-Kpodji (Figure-1). The Plateau department is between 7°10’0 “North Latitude and 2° 34’ 60” East Longitude and covers an area of 3264 km², for about 3% of the national territory with a total population of 622,372 inhabitants [10]. Data were collected in Pobè and Kétou townships in this department (Figure-1).

### Methodology

A survey was conducted on 60 farms, including 35 in Ouémé and 25 in Plateau. The material used is composed of a survey sheet (questionnaire), an electronic scale, and data recording sheets. Information was collected on the breeding mode, the livestock structure, the performed crossbreeding, the reproduction practices, the feeding regime, and the health status of the animals on the farm. Individual data were collected on sows and piglets concerning reproductive performances (number of farrowing, farrowing date, litter size at birth, number of live-born piglets, dead-born piglets, birth-weaning dead piglets, and weaned piglets) and data on sow parents’ origin and on piglets’ growth performances. Information concerning pigs’ origin was also recorded (improved, local, or crossbred). Improved pigs included pigs of exotic breeds and products from their uncontrolled crossings [3]. The exotic breeds included Large White, Landrace, Pietrain and Duroc [3]. Reproductive performances data were collected from 112 litters including 76 of improved sows, 28 from local sows, and 8 from crossbred sows. Piglet weights were recorded during the first 2 months of birth. The piglets were weighed at 0, 30, and 60 days after birth using an automatic scale of 50 kg maximum capacity and an accuracy of ±5 g for weights between 0 and 10 kg, and ±10 g for weights more than 10 kg. The weight data were collected from 44 piglets, including 36 improved, 4 local, and 4 crossbred. The live-born rate, dead-born rate, birth-weaning mortality rate, and weaning rate were determined using the formulas:


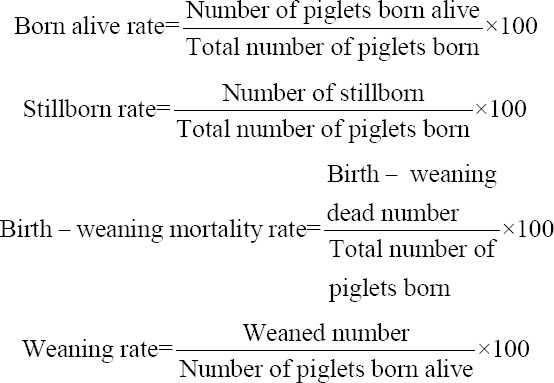


### Statistical analysis

The collected data were analyzed using the SAS software (SAS Institute Inc., Cary, NC, USA) [11]. Concerning the zootechnical performances data analysis, a linear model was adjusted to the data (weight at birth, at 1 and 2 months old, litter size, live-born piglets, dead-born piglets, etc.) and included the fixed effects of the genetic type and the sow origin (internal or external). The interaction between the genetic type and the sow origin was also taken into account in the variance analysis model. This model is as follows:

Y_ijk_=μ+T_i_+O_j_+TO_ij_+ε_ijk_, with:

- Y_ijk_: The zootechnical performance of k pig, of i genetic type, and j origin;
- μ: The general average value;
- T_i_: Fixed effect of i genetic type (local, improved, and crossbred);
- O_j_: Fixed effect of origin (internal or external) of the j sow;
- TO_ij_: Interaction between i genetic type and j origin of the animal;
- ε_ijk_: Random residual effect of k animal, of i genetic type, and j origin.

The generalized linear model procedure (Proc GLM) of SAS was used for the analysis of variance and the averages were then calculated and compared using the t-test.

For the qualitative variables, the frequencies were calculated by the Proc Freq procedure of SAS. Proportions of the two departments were compared by the bilateral Z test. For each relative frequency, a confidence interval of 95% was calculated according to the formula:


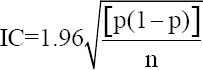


Where, p is the relative frequency and n is the sample size.

## Results

### Profile of the surveyed farms

Pig farmers were mostly men in the departments of Ouémé (91.43%) and Plateau (84%). Women were more involved in Plateau than in Ouémé (16% vs. 8.57%). Investigated farmers were mostly artisans, traders, and breeders in Ouémé, whereas those from Plateau were also involved in crop-production, trades and some were civil servants. In general, farmers of other species than pigs and agro-pastoralists were less common in pig breeding in both departments. Pig breeders were in their majority educated (primary, secondary, or university) in Ouémé (82.86%) and Plateau (68%). The main production objective for all the surveyed breeders was the sale of pig. The other objectives were the use during ceremonies (8-14%), self-consumption (5.71-16%), and savings (0-11.43%). The proportion of breeders with <10 years of breeding experience was significantly higher (p<0.05) in Plateau (72%) than in Ouémé (34%). On the other hand, farmers with 10-20 years of experience in Ouémé (45.7%) were significantly higher than those in Plateau (20%). The animals were mainly bred in total confinement. Nevertheless, some breeders in the Plateau (16%) practiced temporary confinement.

The different types of feed used in farms were complete feeds, raw materials mixtures and kitchens and crop residues. The raw materials mixtures were mainly used (97.14% of breeders in Ouéme and 87.5% in Plateau) and the complete feeds were rarely used (18% in Ouéme and 4% in the Plateau). The raw materials used were oil cakes, cereal bran, and industrial waste. These feeds were distributed twice a day by the majority of breeders in Ouémé (62.86%) and Plateau (70.83%). The rest of breeders from both departments provided feed once or thrice daily. These feeds were supplemented with plants fodder by most of the breeders in Ouémé (60%) and Plateau (80%). The diseases mentioned by the breeders were in order of importance; scabies, diarrhea, anemia, trypanosomosis, ectoparasitic diseases, African swine fever (ASF), gastroenteritis, and mastitis. Diarrhea was more frequently reported (p<0.05) in Ouémé (88.57%) than in Plateau (44%). Mastitis was not reported in Plateau as well as gastroenteritis in Ouémé.

### Structures of pig farms

The pigs used in the two departments were of the local breed, improved breed and the crossing products of these two breeds (Table-1). Improved pigs were more frequently reared (p<0.05) in Ouémé (88.57%) than in Plateau (56%). In contrast, local pigs were more frequently reared (p<0.05) in Plateau (36%) than in Ouémé (8.57%). The number of local boars on the Ouémé farms was higher (p<0.05) than that of the improved boars. The average number of the other categories did not vary significantly between the two departments, apart from the number of local males of age between weaning and reproduction onset that was lower (p<0.05) than that of improved pigs in Plateau (Table-2). The herd size varied on average from 4.67 to 23.89 heads per farm. In Ouémé, the average number of local, crossbred, and improved pigs was 4.67, 18, and 19.45 heads, respectively. In Plateau, the average number of local pigs was 23.89, improved pigs was 20.80, and crossbred was 7.67. The boars’ average numbers ranged from 0.6 to 2.33 heads and those of sows ranged from 1.33 to 4.11 heads.

**Table-1:** Breeds reared in the departments of Ouémé and Plateau.

Breed	Ouémé			Plateau		
	
	n	Percentage	CI	n	Percentage	CI
Local	35	8.57^b^	9.27	25	36^a^	18.82
Improved	35	88.57^a^	10.54	25	56^b^	19.46
Crossbred	35	11.43^a^	10.54	25	20^a^	15.68

CI=Confidence interval, n=Number of breeders surveyed, the percentages of the same row followed by different letters differ significantly at the threshold of 5%

**Table-2:** Livestock structure by breed in the departments of Ouémé and Plateau (Mean±ES).

Variables	Ouémé						Plateau						ANOVA
	
	Local breed (n=3)		Improved breed (n=31)		Crossbreed (n=4)		Local breed (n=9)		Improved brees (n=15)		Crossbreed (n=5)		
					
	Mean	SE	Mean	SE	Mean	SE	Mean	SE	Mean	SE	Mean	SE	
Boar	2.33^a^	0.55	1.00^b^	0.17	1.25^ab^	0.47	1.00^b^	0.32	0.80^b^	0.24	0.60^b^	0.42	*
Sow	1.33^a^	1.49	2.48^a^	0.46	1.50^a^	1.29	4.11^a^	0.86	2.53^a^	0.67	1.80^a^	1.17	NS
Unweaned piglets	0.00^a^	4.35	4.35^a^	1.35	4.25^a^	3.77	5.67^a^	2.51	4.53^a^	1.95	1.20^a^	3.78	NS
Males (Weaning-mating)	0.00^b^	0.32	0.23^b^	0.1	0.25^ab^	0.27	0.00^b^	0.18	0.80^a^	0.14	0.00^b^	0.24	*
Male at fattening	0.67^a^	4.15	5.52^a^	1.29	4.00^a^	3.6	7.78^a^	2.4	5.87^a^	1.86	3.00^a^	3.22	NS
Young male in breeding	0.00^a^	0.5	0.48^a^	0.17	1.25^a^	0.48	0.00^a^	0.32	0.00^a^	0.25	0.00^a^	0.43	NS
Gilt (Weaning-mating)	0.33^a^	0.91	0.94^a^	0.28	2.50^a^	0.79	0.00^a^	0.53	0.93^a^	0.41	0.60^a^	0.71	NS
Gilt at fattening	0.00^a^	3.39	4.16^a^	1.06	2.75^a^	2.44	5.33^a^	1.96	4.87^a^	1.52	1.60^a^	2.63	NS
Young female in breeding	0.00^a^	0.95	0.45^a^	0.3	1.50^a^	0.95	0.00^a^	0.55	0.00^a^	0.43	0.00^a^	1.65	NS
Total livestock	4.67^a^	10.26	19.45^a^	3.19	18.00^a^	8.89	23.89^a^	5.92	20.80^a^	4.59	7.67^a^	17.78	NS

SE=Standard error, NS=p>0.05, **p<0.01, means of the same row followed by different letters differ significantly at the threshold of 5%

### New reproducers supply and breeding model

For reproducers replacement, breeders bought or selected young animals from their own farm (Table-3). Breeders who selected only females by their own farm were the majority (85.29-83.33%) compared to those who selected both sexes by their farms (14.71-16.67%). The separation of future male and female reproducers took place at puberty in 50% of the Ouémé farms and in 42.11% of those of Plateau. However, 44.12% (41.18% before first mating and 2.94% after first mating) and 42.1% (36.84% before first mating and 5.26% after first mating) of the respondents, respectively, in Ouémé and Plateau did not have specific periods for males and females separation. The reproductive male and female were separately housed in all the surveyed farms in Ouémé and in the majority of those of Plateau (100 vs. 76%, p<0.05).

**Table-3:** New reproducers procurement and pig mating in the departments of Ouémé and Plateau.

Variable	Ouémé			Plateau		
	
	n	Percentage	CI	n	Percentage	CI
Reproducers origin						
Selection in the farm	35	97.14^a^	5.52	25	96^a^	7.68
Purchase	35	100^a^	0	25	100^a^	0
Sex of selected animals						
Male and female	34	14.71^a^	11.91	24	16.67^a^	14.91
Female	34	85.29^a^	11.91	24	83.33^a^	14.91
Separation period						
Weaning	34	5.88^a^	7.91	19	15.79^a^	16.40
Puberty	34	50^a^	16.81	19	42.11^a^	22.20
After mating	34	2.94^a^	5.68	19	5.26^a^	10.04
Before mating	34	41.18^a^	16.54	19	36.84^a^	21.69
Separated reproducers lodges						
Yes	35	100^a^	0	25	76^b^	16.74
No	35	0^b^	0	25	24^a^	16.74
Reproducers purchase place						
Farm	30	100^a^	0	15	86.67^b^	17.20
Nigeria	30	0.0^b^	0	15	13.33^a^	17.20
Male and female purchase on the same farm						
Yes	33	30.3^a^	15.68	23	39.13^a^	19.95
No	33	69.7^a^	15.68	23	60.87^a^	19.95
Reproducers purchase from farms where reproducers were sold once						
Yes	34	32.35^a^	15.72	24	33.33^a^	18.86
No	34	67.65^a^	15.72	24	66.67^a^	18.86
Inbred mating						
Animals of the same mother	29	37.93^a^	17.66	22	45.46^a^	28.17
Animals of a family link	29	62.07^a^	17.66	22	54.54^a^	28.17
Reproducers family link						
Brothers and sisters	18	50.0^a^	23.1	14	50^a^	26.19
Cousins	18	11.11^a^	14.52	14	28.57^a^	23.66
Nieces and uncles	18	0.0^a^	0	14	14.29^a^	18.33
Parent and child	18	16.67^a^	17.22	14	50.0^a^	26.19
Filial link	18	44.44^b^	22.96	14	85.71^a^	18.33
Knowledge of consanguinity						
Yes	35	71.43^a^	14.97	25	64.0^a^	18.82
No	35	28.57^a^	14.97	25	36.0^a^	18.82

CI=Confidence interval, n=Number of surveyed breeders, the percentages of the same row followed by different letters differ significantly at the threshold of 5%

For the reproducers purchasing, the majority of breeders from the two departments (93%) obtained animals from neighboring farms. However, in Plateau, some of them bought reproducers from Nigeria. Males and females were bought from the same farm in a percentage of 30.30% in Ouémé and 39.13% in Plateau. Besides, 32.35% of Ouémé and 33.33% of Plateau producers obtain progenitors from farms that have already sold reproducers in the previous years. Matings were performed between animals of the same mother in 37.93% of farms in Ouémé and 45.46% in Plateau. More than half of the pig breeders mated animals having a parental link in both departments (62.07% in Ouémé and 54.54% in Plateau). The majority of Plateau pig breeders (85.71%) and almost half of those in Ouémé (44.44%) had inbred animals of which they did not know the parental link. Among them, the reproducers family link in Ouémé was mainly siblings (50%) or parents-offspring (16.67%). In Plateau, they were siblings (50%), parents-offspring (50%), cousins (28.57%), or nieces and uncles (14.29%) (Table-3). The majority of the breeders in Ouémé (71.43%) and in Plateau (64%) knew the consanguinity effects on the zootechnical performances and the animal health.

### Crossings

The majority of the surveyed breeders performed crossings by themselves. These crossings were more frequent in Ouémé (94.28%) than in Plateau (52%). Most of the breeders did not have a crossing scheme and often crossed improved pigs and crossbred (Table-4). This type of crossing was observed in 87.87% of farms in Ouémé and 69.2% in Plateau. The crossing objectives were the improvement of the zootechnical performances and that of the animal resistance to pathologies. The crossing objective for zootechnical performance improvement was significantly higher in Ouémé (100%) than that in Plateau (76.92%) (p<0.05). Pathologies resistance as crossing objective was reported only in the Plateau (23.08%).

**Table-4:** Crossing between the genetic types reared in Ouémé and Plateau.

Variable	Ouémé			Plateau		
	
	n	%	CI	N	%	CI
Local×Improved pig	33	0.0^b^	0.0	13	15.4^a^	19.62
Local×Crossbreed	33	12.12^a^	11.14	13	15.4^a^	19.62
Improved pig×crossbreed	33	87.87^a^	11.14	13	69.2^a^	25.10
Crossing scheme						
Yes	33	3.03^a^	5.85	13	7.7^a^	14.49
No	33	96.97^a^	5.85	13	92.3^a^	14.49
Crossing objectives						
Zootechnical performances improvement	32	100^a^	0.0	13	76.92^b^	22.90
Resistance to pathologies improvement	32	0.0^b^	0.0	13	23.08^a^	22.90

n=Number, CI=Confidence interval, the percentages of the same row followed by different letters differ significantly at the threshold of 5%

### Consanguinity effect on animals numerical and weight productivity

The consequences of inbred mating affected both reproduction and production performance; high mortality at birth, high birth-weaning mortality, piglets’ weakness at birth, piglets slow growth rate, reduced litter size, reduced live-born number, and high abortion rates. These effects did not differ significantly between departments (Table-5).

**Table-5:** Inbred matings consequences on reproductive performance and piglets’ viability in the farms according to the department.

Variable	Ouémé			Plateau		
	
	n	%	CI	n	%	CI
High mortality at the birth	15	20^a^	20.24	16	25,00^a^	21.22
Birth-weaning high mortality	15	13.33^a^	17.20	16	31.25^a^	22.71
Piglets weakness at the birth	15	33.33^a^	23.86	16	43.75^a^	24.31
Slow growth	15	33.33^a^	23.86	16	62.5^a^	23.72
Reduced litter size	15	53.33^a^	25.25	16	43.75^a^	24.31
Very reduced live-born	15	13.33^a^	17.20	16	6.25^a^	11.86
High abortion rate	15	0^a^	0.00	16	12.5^a^	16.21
Malformation	15	13.33^a^	17.20	16	6.25^a^	11.86

n=Number, CI=Confidence interval, the percentages of the same row followed by same letter do not differ significantly at the threshold of 5%

The litter size at birth was significantly higher (p<0.001) in the improved (8.07) and crossbred (8.25) animals than that of the local breed (6.35). The number of dead-born piglets did not vary significantly among the examined breeds. It was 0.12 for the crossbred, 0.6 for the improved and 0.78 for the local breed (Table-6). From birth to weaning, the number of dead crossbred piglets (2.25) was higher (p<0.05) than that of the improved piglets (0.91). At weaning, the litter size of the improved sows was significantly higher (p<0.05) than that of the local sows. The rates of live-born, dead-born, and birth-weaning mortality and weaned piglets did not significantly vary among the examined breeds (Table-7). Depending on the parent’s origin, the sows born from progenitors originated from other farms had a litter size significantly higher (p<0.001) than sows with parents originated from the same farm (8.49 vs. 6.91). The number of dead-born piglets and birth-weaning dead piglets did not significantly different due to the parents’ origin (Table-8). The dead-born number was 0.49 and 0.72, respectively, for sows of external and internal parents. The number of birth-weaning dead piglets was 0.73 for external parents and 1.30 for internal parents. At weaning, the litter size was higher (p<0.001) for sows with at least one external parent (7.26 vs. 5.05) (Table-8). The rates of live-born, dead-born, and birth-weaning mortality and of weaning of piglets did not significantly vary as a result of the parents’ origin, but sows with at least one external parent’s had higher rates (Table-9). In the local and improved breeds, the birth-weaning piglets’ dead number was significantly higher for sows, of which both parents were from the same farm (Table-10). When calculating the death rate at weaning, this difference became not significant (Table-11). The birth weight was 1.43 kg for piglets from sows with at least one external parent and 1.1 kg for those of sows with both parents born in the farm (Table-12). A month after birth, this weight increases to 4.72 kg for piglets with at least one external parent and 4.09 kg for piglets with all parents of the same farm. The average weight of 60-day-old piglets from sows with at least one external parent was 6.37 kg and 4.74 kg for those of sows with both parents born on the same farm. The piglets’ weight at a typical age did not significantly vary according to the sows’ origin (Table-12).

**Table-6:** Reproductive performances of exploited pig breeds (mean±SE).

Variables	Improved breed (n=76)	Crossbreed (n=8)	Local breed (n=28)	Significance
Litter size	8.07±0.25^a^	8.25±0.79^a^	6.35±0.42^b^	***
Live-born piglets	7.39±0.27^a^	8.12±0.85^a^	5.57±0.45^b^	***
Dead-born piglets	0.60±0.14^a^	0.12±0.44^a^	0.78±0.23^a^	NS
Birth-weaning dead piglets	0.91±0.2^b^	2.25±0.62^a^	0.96±0.33^ab^	*
Weaned piglets	6.66±0.27^a^	5.87±0.80^ab^	4.85±043^b^	***

NS=p>0.05,

* p<0.05,

*** p<0,001, SE=Standard error, means of the same row followed by different letters differ significantly at the threshold of 5%

**Table-7:** Reproductive performances of exploited pig breeds (rate).

Variables	Improved breed (n=76)		Crossbreed (n=8)		Local breed (n=28)	
		
	Rate (%)	CI	Rate (%)	CI	Rate (%)	CI
Live-born piglets	92.51^a^	5.92	98.48^a^	8.46	87.98^a^	12.05
Dead-born piglets	7.49^a^	5.92	1.52^a^	8.46	12.02^a^	12.05
Birth-weaning dead piglets	11.97^a^	7.30	27.69^a^	31.01	8.97^a^	10.59
Weaned piglets	88.03^a^	7.30	72.31^a^	31.01	91.03^a^	10.59

n=Number, CI=Confidence interval, the percentages of the same row followed by same letter do not differ significantly at the threshold of 5%

**Table-8:** Pig reproduction performances according to the parental status (mean±SE).

Variable	External (n=59)	Internal (n=53)	Significance
Litter size	8.49±0.30	6.91±0.29	***
Live-born piglets	8.00±0.32	6.08±0.30	***
Dead-born piglets	0.49±0.17	0.72±0.16	NS
Birth-weaning dead piglets	0.73±0.24	1.30±0.23	NS
Weaned piglets	7.26±0.29	5.05±0.28	***

NS=p>0.05,

*** p<0,001, SE=Standard error

**Table-9:** Pig reproduction performance according to the parental status (rate).

Variables	Internal (n=59)		External (n=53)	
	
	Rate (%)	CI	Rate (%)	CI
Live-born piglets	89.46^a^	7.84	94.22^a^	6.28
Dead-born piglets	10.54^a^	7.84	5.78^a^	6.28
Birth-weaning dead piglets	18.90^a^	9.99	8.96^a^	7.69
Weaned piglets	81.10^a^	9.99	91.04^a^	7.69

n=Number, CI=Confidence interval, the percentages of the same row followed by same letter do not differ significantly at the threshold of 5%

**Table-10:** Interaction between exploited breed and status on the pigs’ numerical productivity (mean±SE).

Variables	Improved breed		Crossbreed		Local breed		Significance
		
	External (n=45)	Internal (n=31)	External (n=4)	Internal (n=4)	External (n=4)	Internal (n=24)	
Litter size	8.64±0.32^a^	7.25±0.39^b^	7.75±1.10^ab^	8.87±1.10^ab^	7.50±1.10^ab^	6.16±0.44^b^	**
Live-born piglets	8.08±0.34^a^	6.38±0.41^bc^	7.50±1.15^ac^	8.75±1.15^ab^	7.50±1.15^ac^	5.25±0.47^c^	**
Dead-born piglets	0.55±0.19^a^	0.67±0.22^a^	0.25±0.63^a^	0.00±0.63^a^	0.00±0.63^a^	0.91±0.26^a^	NS
Birth-weaning dead piglets	0.70±0.26^b^	1.24±0.26^b^	1.25±0.87^ab^	3.25±0.87^a^	0.50±0.87^b^	1.04±0.36^a^	*
Weaned piglets	7.38±0.32^a^	5.48±0.40^bc^	6.25±1.06^ac^	5.50±1.06^ac^	7.00±1.06^ab^	4.47±0.44^c^	**

NS=p>0,05,

* p<0,05,

** p<0,01, ***p<0,001, SE=Standard error, means of the same row followed by different letters differ significantly at the threshold of 5%

**Table-11:** Interaction between exploited race and status on the pigs’ numerical productivity (rate).

Variables	Improved				Crossbreed				Local breed			
		
	External (n=45)	CI	Internal (n=31)	CI	External (n=4)	CI	Internal (n=4)	CI	External (n=4)	CI	Internal (n=24)	CI
Live-born piglets	93.57^a^	7.17	90.67^a^	10.24	96.77^a^	17.33	100^a^	0.00	100^a^	0.00	82.35^a^	15.25
Dead-born piglets	6.43^a^	7.17	9.33^a^	10.24	3.23^a^	17.33	0^a^	0.00	0^a^	0.00	14.38^a^	14.04
Birth-weaning dead piglets	8.79^a^	8.27	18.63^a^	13.71	16.67^a^	36.53	37.14^a^	47.35	6.67^a^	24.45	14.29^a^	14.00
Weaned piglets	91.21^a^	8.27	81.37^a^	13.71	83.33^a^	36.53	62.86^a^	47.35	93.33^a^	24.45	85.71^a^	14.00

n=Number, CI=Confidence interval, the percentages of the same row followed by same letter do not differ significantly at the threshold of 5%

**Table-12:** Piglet growth performance by parents’ provenance (Mean±SE).

Variables	Statut		Significance

	External (n=26)	Internal (n=18)	
W0	1.43±0.12^a^	1.10±0.17^a^	NS
W30	4.72±0.51^a^	4.09±0.94^a^	NS
W60	6.37±0.63^a^	4.74±0.47^a^	NS

NS=p>0.05, SE=Standard error, means of the same row followed by same letter do not differ significantly at the threshold of 5%

## Discussion

### Profile of the surveyed farms

Pig breeding in Ouémé and Plateau is practiced mainly by married men. This result is consistent with that of Djimenou *et al*. [6] and Ognika *et al*. [12] in south Benin and in Congo Republic, respectively. According to Houndonougbo *et al*. [7], the low women involvement in pig breeding slows down this sector development because they can offer improved care of animals by combining small livestock tasks with domestic activities. These farmers belong to all socio-professional groups with a low representation of agricultural producers, as reported by Youssao *et al*. [13] in the local pig farms of Cotonou and Abomey-Calavi peri-urban areas where most of the pig breeders are neither professional breeders nor farmers. Thus, pig breeding is a secondary activity for most of the participants and the sector development needs breeders’ specialization. The pig breeders in both departments have different goals but their major production goal was the sale of pig. This finding shows that animal breeding is a source of income for the surveyed breeders. In addition, other objectives such as savings, self-consumption, and socio-cultural need motivated pig farming in Benin and in West Africa [13,14]. The breeding mode is of improved type. Apart from this mode, the breeders of this locality also practice traditional breeding [5]. The objectives of this study justify the absence of traditional mode because data collection in this mode (characterized by the divagation of animals) is very difficult.

The most widely used feed types in Ouémé and Plateau are raw material mixtures, kitchen, and harvest residues. Very few farmers used the complete feed. The same observations were made by Kiki *et al*. [15]. In addition to these feed resources, Kiki *et al*. [15] reported forage use in pig diets as observed in this study. The majority of the breeders provide feed twice a day in both departments and this is similar to the observation of Kiki *et al*. [15] in the same departments. By contrast, in Douala pig farms, feed is served once a day [12]. The feeding regime reported in this study is related to the types of feed used since, contrary to our study, farmers in Douala peri-urban areas used complete feeds [12]. In the departments of Ouémé and Plateau, animal breeders provide raw materials mixture in the mornings and crop residues and forages in the evenings [15]. The dominant diseases in both departments are scabies, diarrhea, anemia, ectoparasitic diseases, and ASF. These pathologies, especially scabies and ASF, have already been reported in farms in South Benin [13]. These pathologies persistence and spread would be facilitated by the lack of biosecurity measures in the farms. In fact, in the Aguégués farms, breeders most often throw corpses and pigs dejection in the river water and during the floods, this water defiles most of the fodder given to animals [15,16].

### Structures of pig farms

The reared pigs are of local, improved, and crossbred genetic types. These genetic types have been reported in pig farms in South Benin [3]. Improved pigs are more exploited. This dominance could be explained by the breeders’ main objective which is pigs’ production for sale. Improved pigs, for example, have higher zootechnical performances than local and crossbred pigs [1,3] and would give breeders the best returns from their fattening. For all breeds, the average pigs’ number per farm ranged from 4.67 to 23.89 heads in both departments. This number is close to 10-23 heads reported by Djimenou *et al*. [6] in south Benin. The sows average numbers of 1.33-4.11 heads obtained in this study are close to 2-4 sows reported in pigs farms in Benin [6,7,13] and to 3.9 sows in Bangui (Central African Republic) [17]. The number of boars varies from 0.6 to 2.33 heads and these numbers are similar to 0-3 boars reported by Youssao *et al*. [13] and Houndonougbo *et al*. [7]. Some farms do not have reproductive boars and this is justified by the farms’ small size and the high maintaining cost of a boar [7,13]. For animals mating, these breeders are obliged to borrow boars from neighboring farms [7,13]. This practice would promote consanguinity in the farms because the same male is used on several farms and breeders sell reproducers within each other.

### Zootechnical performances improvement and consanguinity management

The majority of breeders select reproductive pigs from their own farm. The selection of male and female progenitors from the same farm is a factor favoring inbred mating, especially since kinship links are rarely included in the reproductive male selection criteria [5]. The criteria for choosing males in Ouémé and Plateau are conformation, health status, absence of genetic defects, testicular development, teats number, and piglet growth; those used for females selection are litter size, piglet growth, health status, teats number, and maternal behavior [5]. This last criterion must also be taken into account in the males’ choice. The future reproducers separation is done at puberty and animals are then housed separately by most of the breeders. The group housing of weaned piglets improves the age at puberty [18]. The piglets separation at puberty permits breeders to avoid inbred matings because they were separated when they were able to mate. Contrary to this practice, the renewal animals purchase in farms to which they had already sold reproducers promote inbreeding. Thus, the creation of breeding farms for selection is indispensable. These farms will not only reduce the consanguinity effects on farms but they will also improve the zootechnical performances by enabling an improved choice. The majority of the surveyed breeders perform inbred matings, and this practice is due to the increased use of animals of high performance favored by the small herds’ size. Thus, it is difficult for breeders to separate them from animals of high performance and the male maintenance for a long time on the farm favors inbred matings, especially because the same male is sometimes used in several farms. Similar practices have been reported in Madagascar [19], but these practices lead to a decrease in performance instead of improvement. The majority of the surveyed breeders perform crossings, but none has a specific crossing model. The same observations were made by Youssao [2] in pigs’ farms in Benin, and this is mainly due to the lack of monitoring and control in the farms. Indeed, animal breeding services should monitor and validate all crossing made by farmers. The crossings objective is especially to improve, first the zootechnical performances and then the pathologies resistance. This justifies the priority choice of improved pigs by breeders because of their zootechnical performances. These breeders cross males of this genetic type with crossbred sows having already received the resistance to pathologies from their local parents by complementarity.

### Consanguinity effect on the zootechnical performance

The consanguinity consequences according to the breeders are the high mortality at the birth, the high birth-weaning mortality, the piglets’ weakness at the birth, the slow growth rate, the reduced litter size, and the high abortion rates. The consanguinity as cause of reproduction and growth performances decrease has already been reported in pigs [8,20,21]. Thus, consanguinity reduces performances particularly the litter size, the piglet weight, the age at puberty, and the boar libido [9,22]. The genes responsible for the depression of litter size and number of live births in sows are carried by the chromosome 13 in the 27-54 Mb regions and are *inter-alpha-trypsin inhibitor* genes groups involved in the implantation of the embryo [22]. Indeed, the trophoblast secrete a *trypsin-like* protease, which facilitates embryo implantation in the endometrium [23]. The consanguinity rate is higher in small farms such as those in this study than in large farms [18]. To reduce these effects, farmers must increase the herd size, especially the number of males. Animals with all parents from the same farm performed lower than animals with at least one parent from an external farm. This observation shows that it could be a parental link between these animals. This kinship link existence which causes animals low performances is confirmed by the breeders whose majority has recognized to perform inbred matings. These matings must be avoided by the breeders because they lead to a decrease in reproductive performances [9].

## Conclusion

The study on crossing and consanguinity management in pig farms of Ouémé and Plateau shows that the reproduction practices implemented do not preserve farms from consanguinity. Thus, some breeders select reproductive males and females from their own farms, while others buy them from farms where they have sold reproducers in the past. Breeders cross inbred animals. They mate crossbred pigs with improved pigs without any crossing scheme. These breeders are well aware of the consanguinity consequences of these inbred mating but do so aiming to improve the zootechnical performances of reared pigs. Sows from external farms had higher zootechnical performances than sows from parents born on the farm. In view of the high consanguinity risk in our farms, it is indispensable to evaluate the zootechnical performances of the reared pigs.

## Authors’ Contributions

IOD, SF, IYAK, and NA conceived the study design. IOD, SI, PK, and BG collected the data. GAM, BK, and IYAK analyzed the data. IOD and SI wrote the manuscript. MD, JD, and PT corrected the manuscript. All authors read and approved the final manuscript.
